# Evaluation of teaching effect of first-aid comprehensive simulation-based education in clinical medical students

**DOI:** 10.3389/fpubh.2022.909889

**Published:** 2022-08-10

**Authors:** Mian Peng, Ning Su, Rui Hou, Huijuan Geng, Fangfang Cai, Weixiong Zhong, Weifang Zhang, Jingxing Zhong, Zhengyue Yang, Weiling Cao

**Affiliations:** ^1^Department of Critical Care Medicine, The Third Affiliated Hospital of Shenzhen University, Shenzhen, China; ^2^Department of Pharmacy, The Third Affiliated Hospital of Shenzhen University, Shenzhen, China

**Keywords:** first aid, comprehensive, simulation-based education, clinical medical students, teaching

## Abstract

**Background:**

Although students mastered the composition skills, they lack of the ability to effectively integrate these composition skills in real clinical situations. To address the problem, we set up different levels of situational simulation training for medical students in grades 2–4, and evaluate the teaching effect of first-aid situation comprehensive simulation-based education (SBE) on clinical medical students.

**Methods:**

The medical students in Grade 2, 3, and 4 received different situational SBE, respectively. The 2nd-year medical students received a single skill module which included cardiopulmonary resuscitation, endotracheal intubation, and electric defibrillation training. The 3rd-year medical students received a single subject module which included cardiovascular and respiratory system training. The 4th-year medical students received the integrated multidisciplinary module which combined first-aid skills, clinical thinking, and teamwork training. The primary outcome was the expert evaluation and peer evaluation. The secondary outcome was students' satisfaction questionnaire response. In our training, we arranged an adequate teaching staff for intensive training and timely feedback (the student–teacher ratio of 5:1), adequate time for repetitive practice (Each SBE was carried out within 4 h), curriculum design, and integration from real cases by clinicians, realistic computer-driven mannequins to ensure simulation fidelity, providing a different difficult level of SBE to different grades of students, and pre- and post-tests for outcome measurement.

**Results:**

In all of the single skill module, single subject module or comprehensive disciplines module, the scores in the expert evaluation and peer assessment after the training were significantly higher than before the training, and the differences were statistically significant (*p* < 0.05). The integrated subject training, although having the lowest pre—and post-test marks, had the largest increase in score.

**Conclusion:**

The first aid comprehensive simulation-based education in grade 2–4 clinical medical students, basing on timely feedback, repetitive practice, curriculum integration, simulation fidelity, and outcome measurement are effective in improving the students' proficiency in managing the real emergencies.

## Background

Simulation has had a long and varied history in many different fields, such as aviation and the military. Healthcare, like aviation, is driven by safety, more specifically patient safety. Clinical medicine has begun to focus on the safety of patients and the quality of their practice and not merely on medical education, as well as, patients are paying more attention to the “practice” from medical students and residents on them (1, 2). In 1999, Anderson M et al. reported that many observers of medicine have expressed concerns that new doctors are not as well–prepared as they should be to meet society's expectations of them (3). At present, with the increasing of medical information and research, educators need to constantly adjust the curriculum structure to improve the teaching effective and meet the strict teaching requirements. Therefore, developing medical simulation teaching is more important for improving clinical practice ability. In the history of medical education, the earliest simulated teaching method originated from anatomy. The rise of learning, with the continuous expansion of medical teaching content and the improvement of modern manufacturing technology and electronic information technology, medical simulation technology is becoming more and more perfect in terms of functionality and simulation. As the link between simulation and patient safety becomes increasingly apparent, simulation was adopted as the education and training method of choice for such critical behaviors as communication and teamwork skills worldwide (4).

The classroom model remains disconnected from the actual clinical environment. Many students also lack of training in theoretical study, physical examination, and diagnosis (5). In China, the clinical skills training courses, which appeared in 2000, have experienced from the pure practice of basic skills on simple models to elementary simulation courses, preliminary solving the problems of dispersion of basic skills training and lack of practice chance. Since 2010, the medical college clinical skills centers have kept expanding. The skills training equipment has developed from simple models to computer-driven high simulation models. At the same time, the training and use of standardized patients (SPs) have also kept increasing, which greatly improved the quality of clinical skills training (6). However, the rapid improvement of hardware and SPs also increased the demands for the development of situational comprehensive simulation clinical skills training courses, aiming at improving students' clinical logic reasoning, practice ability, and teamwork.

Medical education attaches great importance to practical operation, and qualified doctors cannot be trained without clinical practice. The traditional teaching model has encountered difficulties in adapting to the requirements of modern teaching, and the quality of clinical practice teaching has been greatly affected, which restricts the development of medical clinical teaching (7). The application of medical simulation teaching, which is more in line with humanistic care, is becoming the dominant mode of clinical medical practice teaching, and is more and more favored by clinical medical education (8). In the course of teaching, we found that although after the early stage of training, medical students have mastered some clinical skills. However, in clinical practice, they still cannot meet the teaching demands of clinical application of basic skills. This is to say, although students mastered the composition skills, they lack of the ability to effectively integrate these composition skills. They are unable to effectively apply the component skills in real clinical situations.

To address these problems, it is very urgent to construct the first aid simulation-based education (SBE) scenarios and to perform comprehensive simulation teaching, and equally importantly, to evaluate the teaching effects (9). Thus, we set up different levels of situational simulation training for Grade 2–4 medical students, and evaluate the teaching effect of first aid situation comprehensive SBE on clinical medical students.

## Methods

An experiment was conducted to evaluate the effect of SBE on the knowledge, self-efficacy improvement, and composite scores of clinical students.

### Participants

The full-time medical students in Grade 2, 3, and 4 of clinical medicine in Shantou University Medical College were eligible for admission to the study. The total number of grade 2 students in Shenzhen class is 35. Excluding those who have received first aid (such as cardiopulmonary resuscitation, endotracheal intubation, and electric defibrillation), 30 students are enrolled. There are a total of 34 students in Grade 3 Shenzhen class, 4 students who have received first aid training were excluded. There are 35 students in Shenzhen class of grade 4. Excluding those who have received first aid training, 30 students are enrolled. The study was undertaken from September 1 to December 31 in 2021. The written consent was obtained prior to the commencement of the scenario. The Ethics Approval was obtained from the Ethics Committee of the third Affiliated Hospital of Shenzhen University.

### Study procedure

**Figure 1:**
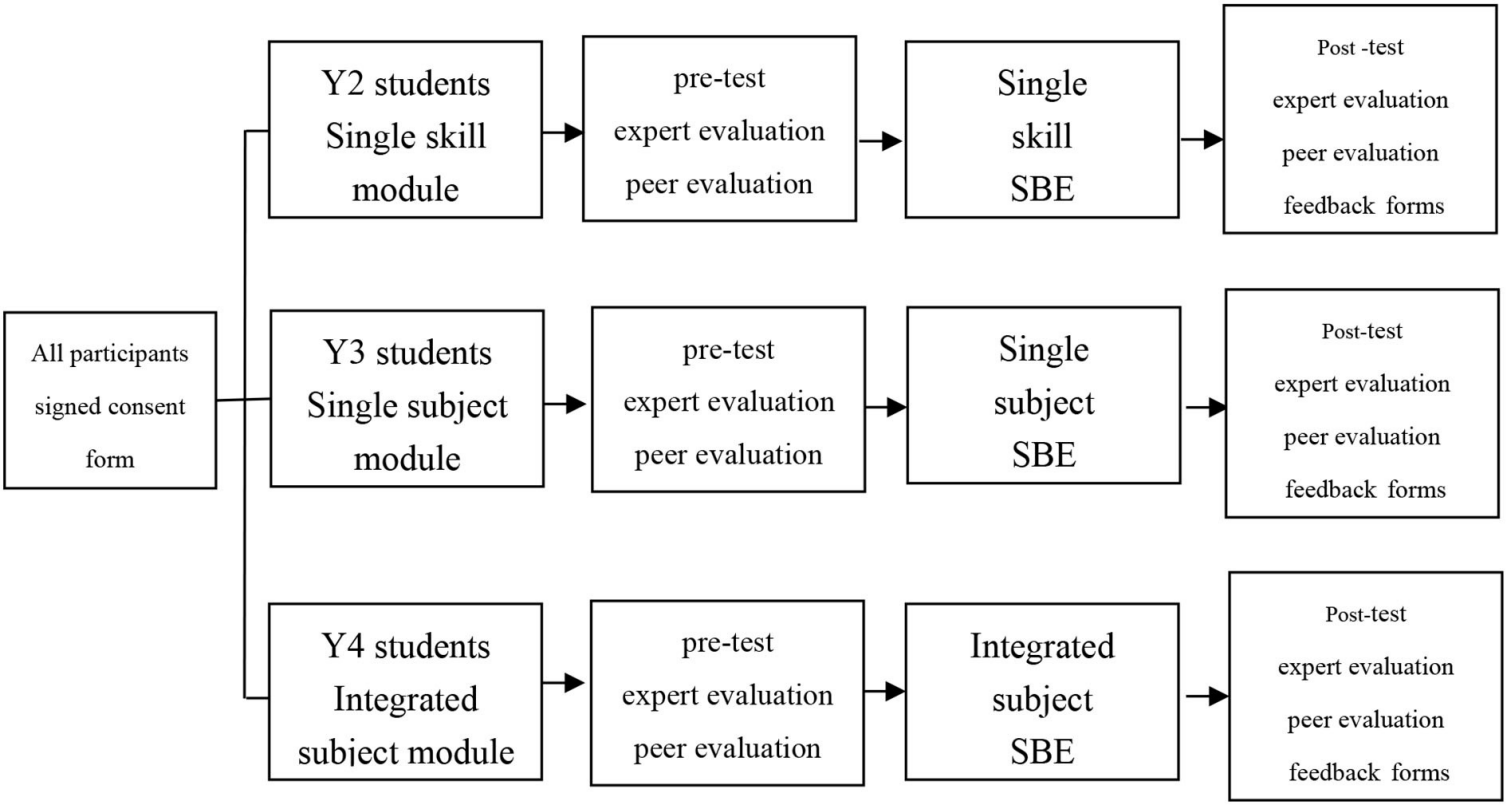
The general flowchart of the whole research. SBE, simulation-based education.

The medical students in Grade 2, 3, and 4 received different situational SBE, respectively. The design and implementation of the first aid SBE for each grade were carried out by the teachers from the Department of Critical Care Medicine of the Third Affiliated Hospital of Shenzhen University. For the 2nd-year medical students, the SBE is a single skill module which included cardiopulmonary resuscitation, endotracheal intubation, and electric defibrillation training. For the 3rd-year medical students, the SBE is a single subject module that includes cardiovascular and respiratory system training. For the 4th-year medical students, the SBE is the integrated multidisciplinary module that combined first-aid skills, clinical thinking, and teamwork training. The study procedure was listed in Figure 1.

The mannequins used in SBE are realistic, anatomically correct, and computer-driven, which can simulate the physiological reactions of real patients. Each SBE was carried out within 4 h, with 30 students in each grade. The student–teacher ratio is 5:1.

### Outcome measures

The primary outcome measured in this research project was expert evaluation and peer evaluation. The expert evaluation was performed through the expert evaluation table. Peer evaluation was performed through the peer evaluation form. The students' satisfaction with SBE was the secondary outcome of this project and was measured with training feedback forms. The questionnaire was measured on a 5-point Likert scale ranging from 0 (Not at all) to 4 (Totally).

In the first 5 min of each class, all students who participated in these classes were required to take a pre-training test, and the expert evaluation form and peer evaluation form were required to complete. All of these forms were collected before the beginning of each training. At the end of the training courses, the students were required to take a post-training test and the same two forms, together with the students' own learning feedback forms. All questionnaires were issued in paper forms.

### Statistical analysis

All analyses were performed by using SPSS version 22 (IBM Inc., New York, United States). All statistical tests were 2-sided, and *p*-value < 0.05 indicated statistical significance. An independent *t* test was performed to compare the continuous variables of the normal distribution, and a Mann–Whitney *U* test was performed to compare the continuous variables of skew distribution. The scores were expressed as means plus or minus standard deviations (*SD*).

## Results

For the cohort of 90 medical students enrolled in the study, none was excluded or declined to participate. Questionnaire response rates for each of the three SBE scenarios and learning reactionnaire are 100%.

In all of the single skill SBE cardiopulmonary resuscitation, endotracheal intubation, electric defibrillation, the single subject SBE (cardiovascular, respiratory), or the comprehensive disciplines SBE, the scores in the expert evaluation and peer assessment after the training were significantly higher than before the training, and the differences were statistically significant (*p* < 0.05, see Table 1). The highest scores pre- and post-test were in the single-skill SBE, and the lowest scores pre- and post-test were in the comprehensive subject SBE.

**Table 1:** Change in expert evaluation, peer evaluation, and learning reactionnaire response after each simulation-based education (SBE).

**‘Grade**	**SBE**	**Expert evaluation**				**Peer evaluation**				**Learning reactionnaire reponse**		**Total**
		**Mean pre score**	**Mean post score**	**Mean difference**	***P*-value**	**Mean pre score**	**Mean post score**	**Mean difference**	***P*-value**	**Rank ≥3(%)**	**Rank ≥2(%)**	
Grade 2	Single skill 1	53.4 ± 16.5	71.7 ± 14.4	18.3	<0.001	83.1 ± 7.3	90.2 ± 6.9	7.1	<0.001	93.3	100	30
	Single skill 2	46.7 ± 22.1	69.5 ± 24.6	22.8	<0.001	53.4 ± 28.0	82.4 ± 8.7	29	<0.001	93.3	96.7	30
	Single skill 3	49.3 ± 15.8	84.6 ± 10.4	35.3	<0.001	52.5 ± 16.6	84.4 ± 15.0	31.9	<0.001	93.3	96.7	30
Grade 3	Single subject 1	50.6 ± 15.4	73.9 ± 4.3	23.3	<0.001	58.1 ± 11.0	76.2 ± 4.7	18.1	<0.001	90	96.7	30
	Single subject 2	48.2 ± 12.6	62.8 ± 11.5	14.6	<0.001	59.2 ± 10.7	72.0 ± 5.9	12.8	<0.001	96.7	100	30
Grade 4	Integrated subject	28.0 ± 14.3	58.3 ± 7.2	30.3	<0.001	39.9 ± 21.1	62.0 ± 13.9	22.1	<0.001	96.7	100	30

skill 1, cardiopulmonary resuscitation; skill 2, endotracheal intubation; skill 3, electric defibrillation.

subject 1, cardiovascular system training; subject 2, respiratory system training.

There was a 100% response rate to the learning reactionnaire. The results indicated that 93.3% of the students scored ≥ 3 (A lot), and 100, 96.7, 96.7, 96.7, 100, and 100%, of the students scored ≥ 2 (Moderately) (see Table 1), indicating that the medical students had a positive feedback on the training effect of SBE.

A total of 90 students from the SBE group provided their complete feedback on the SBE (Table 2). For this course, 95.5% of the students were satisfied with our SBE and more than 80% have a good evaluation (evaluation of the necessity, 95.5%; evaluation of the form, 94.8%; evaluation of the schedule, 81.4%; level of participation, 95.5%). The majority of these students also thought that SBE may help them improve themselves effectively (98.9%). Moreover, 97.8% of the students were satisfied with their teacher.

**Table 2:** The feedback of students in the SBE group.

**Feedback items**	**0 point**	**1 point**	**2 point**	**3 point**	**4 point**
	**Very disagree/dissatisfied Very agree/satisfied**				
My overall satisfaction with this SBE course	0.0%	0.0%	4.5%	49.2%	46.3%
Evaluation of the necessity of this SBE course	0.0%	0.0%	4.5%	38.4%	57.1%
Evaluation of the form of this SBE course	0.0%	1.1%	5.1%	46.9%	46.9%
Evaluation of the schedule of this SBE course	0.6%	4.0%	14.1%	46.9%	34.5%
My level of participation in the process of this course	0.0%	0.6%	4.0%	55.4%	40.1%
SBE course helps me better grasp the clinical operation	0.0%	0.0%	1.1%	42.4%	56.5%
My satisfaction with the teachers of SBE course	0.0%	0.6%	1.7%	32.8%	65.0%

## Discussion

Medical education is rapidly changing, influenced by many factors including the changing healthcare environment, the changing role of the physician, altered societal expectations, rapidly changing medical science, and the diversity of pedagogical techniques (10). The traditional teaching method is faced with many problems such as fewer clinical patients and uncooperative patients; compared with traditional teaching methods, the advantages of simulation-based education are step-by-step learning, a “hands-on learning experience and the opportunities for repetition until proficiency is reached” (11–13). At present, the first-aid training for clinical 2–4 grade students in medical colleges in China is only in the traditional mode of theoretical teaching by teachers and skills training on models. This teaching method makes medical students lack of clinical thinking, team cooperation, and humanistic concepts. Without the training of various clinical situations, medical students, even after such training, often do not know what to do when they meet real patients in clinical practice, especially in critically ill patients (14).

In real situations, with the development of society, the enhancement of the legal awareness of the whole people and the clear right to know, consent and privacy of patients, as the subject of traditional teaching, patients have the right to refuse to be the object of teaching, especially the examination of some private parts. Most patients refuse to cooperate with the teaching (15). This makes clinical teaching often into an awkward situation. Moreover, the use of real patients in clinical teaching may lead to medical disputes, so that clinical teaching hospitals often sacrifice teaching, medical students should master the various clinical skills which cannot be properly trained (14). According to China's “Medical Practitioners Law”, medical students can only be legally engaged in medical practice after obtaining a medical qualification and at the side of patients and their families, especially critically ill patients, they do not cooperate with clinical teaching and refuse to be managed by interns (11). At the side of clinical teachers, they do not arrange interns to deal with critically ill patients due to concerns about medical disputes. In the present course of medical students' skill training, the attention is only paid to the cultivation of composition skills, without systematic logical reasoning, multidisciplinary cooperation, and development of emergency management ability to deal with critically ill patients (16).

In order to solve these problems, it is very urgent to construct first-aid simulation scenarios and conduct comprehensive SBE on this basis, The prevailing view is that SBE is superior to traditional teaching methods (17, 18). In medical education, the traditional education model is generally that students listen to their teacher, and then learn knowledge through their own understanding or observation. The traditional education model fails to provide simulated learning opportunities that affect their transition from theory to practice (19). SBE is also an increasingly important educational strategy that plays an important role in improving patient safety. SBE trainees can improve professionally by learning from their mistakes, which will help them avoid the same mistakes in real life. The main motivation for this study was to evaluate the effectiveness of SBE in training undergraduate students. A study with a sample size of 1,178 students suggested that SBE could have a positive impact on college education. Students in the SBE group had more positive communication with classmates and teachers, which could explain their great improvement in humanistic care and doctor–patient communication (20).

**Figure 2:**
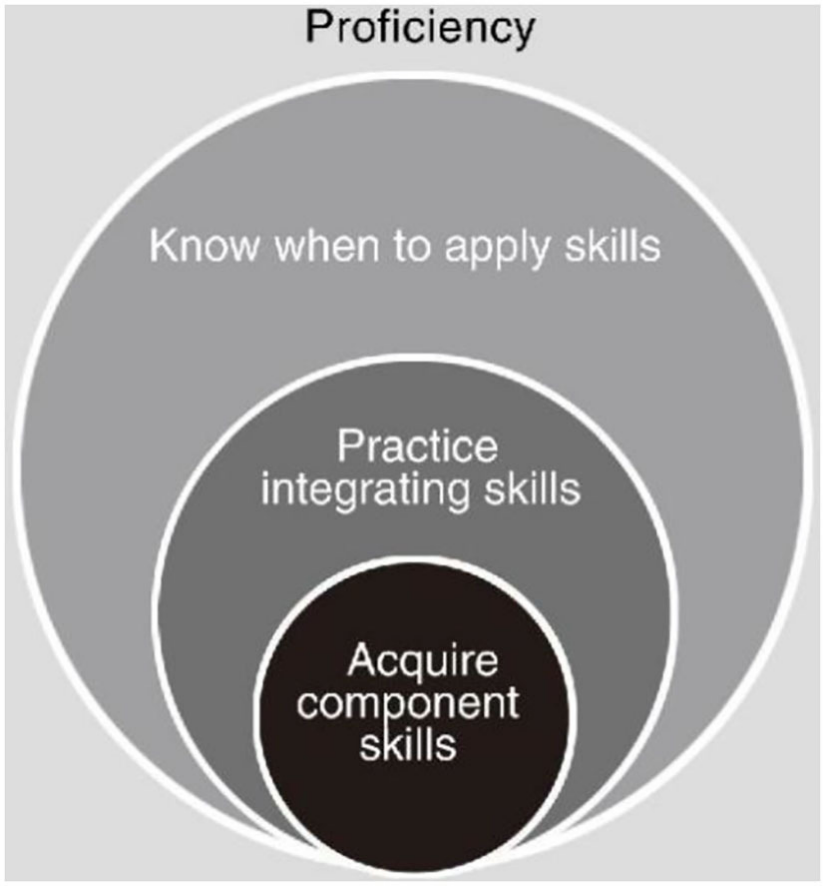
The ingredients of proficiency.

The purpose of the simulation is to imitate real patients, clinical tasks, and the real environment provided by medical services. It can provide feedback after simulation, with repetitive practice habits, course integration, and appropriate difficulty level. The researchers point it out that in order to achieve proficiency, students firstly need to acquire the component skills, then practice to integrating them, and proceed to know when to apply the skills they have learned (Figure 2) (21). The significance of simulation teaching lies not only in the application of simulated mannequins, but also in the integration of clinical situations. It is very important to integrate the clinical context into the teaching scenarios. First of all, the single skill training teaches only component skill, resulting in students' deficiency of clinical thinking. Secondly, without the experience of simulated scenarios, medical students often do not know how to deal with real clinical situations. Thirdly, the integration of clinical situation is helpful to train the medical students' teamwork, humanistic spirit, and to lead students to systematically integrate the knowledge and skills they have learned, thus helping them to reach the level of proficiency (22, 23).

Therefore, we set up three levels of situation simulation for medical students in grades 2, 3, and 4, and evaluate the teaching effect of first-aid comprehensive simulation-based education in these clinical medical students. Our study showed that whether in the single skill SBE [cardiopulmonary resuscitation (CPR), endotracheal intubation, electric defibrillation], the single subject SBE (cardiovascular, respiratory, or the comprehensive disciplines SBE, the scores in the expert evaluation and peer assessment after training were significantly higher than scores before training, and the differences were statistically significant (see Table 1). Our study is consistent with the reports of Issenberg SB et al. (24) and McGaghie WC et al. (25), which showed that SBE with deliberate practice is superior to traditional clinical medical education in achieving specific clinical skill acquisition goals, especially when the courses were arranged with different difficult levels, good curriculum integration, timely feedback, and repetitive practice. We have also developed a feedback questionnaire (a specific evaluation of the course). Feedback is a major component of reporting. This two-way interactive discussion can help participants reflect on their behavior and performance. Feedback is the primary component of debriefing. This bidirectional and interactive discussion could help participants reflect on their actions and performances (26).

Our results also show that while the integrated subject SBE scored lowest in pre- and post-school tests, there was a significant increase in scores. Our integrated clinical situations are modified according to real cases by clinicians, which can simulate the clinical situations to the greatest extent. Not only can we provide medical students with very realistic clinical situations, but also the training is very safe and effective. Medical students often lack confidence in handling common clinical emergencies. Morris MC et al. (25) designed a new sub-module to incorporate high-fidelity simulation into the undergraduate medical curriculum and found that students showed a very positive attitude toward this new teaching method, especially in integrating previously learned knowledge and skills, indicating that simulation-based teaching is feasible and effective in the undergraduate setting.

Among the 2nd-year medical students' single skill training, the skill of electrical defibrillation increased the most after training, and the score of endotracheal intubation increased the least after training. This may be due to the relatively few training steps for electric defibrillation and the complexity steps for endotracheal intubation. Therefore, we need to increase theoretical learning of endotracheal intubation and increase the number of trainings to better master this clinical skill.

Simulation-based medical education is a complex service intervention that needs to be planned and practiced with attention to organizational contexts. In our study, we arranged an adequate teaching staff for intensive training and timely feedback (the student–teacher ratio of 5:1), adequate time for repetitive practice (each SBE was carried out within 4 h), curriculum design and integration from real cases by clinicians, realistic computer-driven mannequins to ensure simulation fidelity, providing different difficult level of SBE to different grades of students, and pre- and post-tests for outcome measurement. These factors are consistent with the features and best practices of SBE that is convinced to be used in medical simulation technology to maximum educational benefits (27). Other studies have also shown that medical simulation teaching can provide opportunities and conditions for medical students to contact the clinical practice in the early stage of school, which can improve medical students' clinical skills, operation ability, bedside comprehensive diagnostic thinking ability, and is beneficial to the cultivation of students' professional ethics and code of conduct (28, 29). Simulation teaching advocates medical teaching in a way that is as close to the real clinical environment as possible and more in line with medical ethics, and uses all simulated hand segments to create simulated patients, simulated scenes, simulated classrooms for various skills training and assessment, simulated wards, simulated operating rooms, and other software and hardware conditions (13). As an effective supplement to theoretical teaching and clinical practice, simulation teaching has changed the traditional teaching model, provided a safe teaching environment, cultivated agile and correct clinical thinking, and reduced the occurrence of medical accidents and disputes in clinical practices (30).

Our study has limitations. First, only 30 medical students of each grade were included. More participants are needed to further confirm the effectiveness of the first-aid-integrated comprehensive SBE. Second, although improvements were observed in our study post-training when compared with pre-training; there is no comparison to standard educational methods. Thirdly, this research was only based on a single center. Only grade 2–4 medical students were included, so the conclusion cannot be generalized to other populations. Finally, the participants are all from the Shantou University Medical College and may not represent students from other medical colleges.

## Conclusion

The first-aid comprehensive simulation-based education in grade 2–4 clinical medical students, basing on timely feedback, repetitive practice, curriculum integration, simulation fidelity, and outcome measurement are effective in improving the students' proficiency in managing the real emergencies.

## Data availability statement

The raw data supporting the conclusions of this article will be made available by the authors, without undue reservation.

## Ethics statement

The studies involving human participants were reviewed and approved by Shenzhen Luohu People's Hospital. The patients/participants provided their written informed consent to participate in this study.

## Author contributions

MP and NS prepared the tables and drafted the manuscript. RH, HG, FC, WZho, WZha, JZ, and ZY reviewed the manuscript for its intellectual content. WC were responsible for revising the manuscript. All authors have read and approved the final manuscript.

## Funding

This study was supported by the clinical teaching reform research project from the Education Office of Guangdong Province in 2019 (2019 JD115). This article is a summary of the research project.

## Conflict of interest

The authors declare that the research was conducted in the absence of any commercial or financial relationships that could be construed as a potential conflict of interest.

## Publisher's note

All claims expressed in this article are solely those of the authors and do not necessarily represent those of their affiliated organizations, or those of the publisher, the editors and the reviewers. Any product that may be evaluated in this article, or claim that may be made by its manufacturer, is not guaranteed or endorsed by the publisher.
